# Genetic Diversity of Promising Spring Wheat Accessions from Russia and Kazakhstan for Rust Resistance

**DOI:** 10.3390/plants13172469

**Published:** 2024-09-04

**Authors:** Elena Gultyaeva, Ekaterina Shaydayuk, Ekaterina Shreyder, Igor Kushnirenko, Vladimir Shamanin

**Affiliations:** 1 All Russian Institute of Plant Protection, Shosse Podbelskogo 3, 196608 St. Petersburg, Russia; eshaydayuk@bk.ru; 2 Chelyabinsk Scientific Research Institute of Agriculture, 456404 Timiryazevskiy, Russiaikush2001@mail.ru (I.K.); 3 Department of Agrotechnology, Omsk State Agrarian University, 644008 Omsk, Russia; vp.shamanin@omgau.org

**Keywords:** molecular markers, *Puccinia graminis*, *Puccinia striiformis*, *Puccinia triticina*, resistance, resistance genes, *Triticum aestivum*

## Abstract

Spring bread wheat (*Triticum aestivum*) is a major crop in Russia and in Kazakhstan. The rust pathogens, leaf rust caused by the fungus *Puccinia triticina*, stem rust incited by *P. graminis* and yellow rust caused by *P. striiformis*, are the significant biotic factors affecting wheat production. In this study, 40 new promising spring wheat genotypes from the Kazakhstan-Siberia Network for Spring Wheat Improvement (KASIB) were tested for resistance to leaf, stem and yellow rust at the seedling stage, and for identification of rust resistance genes using molecular markers. In addition, the collection was tested for leaf rust resistance and grain yields in the South Urals agroclimatic zone of Russia in 2023. As a result, 16 accessions with seedling resistance to leaf rust, 21 to stem rust and 4 to yellow rust were identified. Three breeding accessions were resistant to all rust species, and nine to *P. triticina* and *P. graminis*. Wheat accessions resistant to leaf rust at the seedling stage were also resistant in the field. Molecular analysis showed the presence of cataloged resistance genes, *Lr1*, *Lr3a*, *Lr9*, *Lr10*, *Lr19*, *Lr20*, *Lr24*, *Lr26*, *Sr15*, *Sr24*, *Sr25*, *Sr31*, *Sr38*, *Yr9* and *Yr18*; uncatalogued genes *Lr6Agi1* and *Lr6Agi2* from *Thinopyrum intermedium* and *LrAsp* from *Aegilops speltoides*; and 1AL.1RS translocation. The current analysis showed an increase in leaf and stem rust resistance of new KASIB genotypes and their genetic diversity due to the inclusion of alien genetic material in breeding.

## 1. Introduction

Spring bread wheat (*Triticum aestivum*) is a major crop in Russia and Kazakhstan. In Russia, spring wheat is grown in the Volga region, Western Siberia and the Urals [1]. In Kazakhstan, it is predominant in the northern regions [2]. The rust pathogens, leaf rust (causal agent *Puccinia triticina*), stem rust (causal agent *P. graminis*) and yellow (stripe) rust (causal agent *P. striiformis*), are the significant biotic factors for wheat production. Long-distance dispersal capacity, rapid changes in virulence and climatic adaptability make wheat rusts the most important threat to wheat production worldwide [3]. Of the three rust species, *P. triticina* is the most widely spread pathogen and develops annually in spring wheat areas in Russia and Kazakhstan. For a long time, *P. graminis* was not widely distributed, but in recent years, its importance has increased [4,5]. In Western Siberia in 2016–2018, strong development of stem rust led to a 25–35% reduction in grain yields [5]. *P. striiformis* is not a common disease of spring wheat in Russia and Kazakhstan. Nevertheless, it has been observed in West Siberia since 2015 [6]. In Kazakhstan, the main zone of yellow rust distribution is in the southern and southeastern regions [7]. However, its high dispersal capacity may contribute to the introduction of this pathogen to the North Kazakhstan regions, and under favorable weather conditions, this may lead to the development of the disease. A range of effective fungicides are available, but host resistance remains the most ecologically sustainable method for rust control. For this reason, the determination and evaluation of the presence of wheat cultivars resistant to rust diseases is of great importance for breeding. The durability of resistance and genetic diversity of wheat genotypes are the most important features of the resistance for global wheat improvement programs. Therefore, it is important to identify resistance genes present in promising wheat lines and commercial cultivars, as they may share resistance genes even if their pedigrees are different. Knowledge of resistance genes can prevent the release of monogenic cultivars containing the same resistance genes [8,9].

In the late 1990s, the International Maize and Wheat Improvement Center (CIMMYT) began a collaborative research and breeding program with northern Kazakhstan and Western Siberia, and by 2000, it had evolved into the Kazakhstan-Siberia Network for Spring Wheat Improvement (KASIB). The breeding institutions that contributed material stretch from the Volga region to the Ural Mountains, Western and Eastern Siberia in Russia, and northern, central and southern parts of Kazakhstan [2]. This has enabled the evaluation of the grain yield, quality, disease resistance and other traits through cooperative multi-location trials using materials exchanged between leading research and breeding institutes in both countries. Shuttle breeding with CIMMYT was used to incorporate stem and leaf rust resistance while maintaining local adaptation, drought tolerance and grain quality. Two to four new and promising genotypes are supplied by participating organizations and tested over 2 years by all cooperators. This program is an exemplary mechanism for germplasm evaluation and disease monitoring [2]. Notably, the State Registers of Breeding Achievements have included many elite genotypes from the program for commercial production in Russia and Kazakhstan.

Along with field trials, the program involves seedling stage assessment in the greenhouse using pathotypes with established virulence attributes. Molecular markers are used to identify rust resistance genes. Being complementary and confirmatory, the effectiveness of screening seedlings and adult plants and the use of diagnostic markers has been greatly enhanced.

With leaf rust being an ongoing problem for the production of spring wheat in Russia and Kazakhstan up to the mid-2010s, research and breeding institutes gave priority to developing new resistant cultivars, as there was initially a low proportion of resistant material identified [2]. With time, some progress has been achieved and the dynamics of increasing resistant leaf rust genotypes in the KASIB network remains stable in 2020 [10,11,12]. Due to the increasing potential importance of other rust species, it is relevant to expand research on the resistance of KASIB material to other rust species and to assess their genetic diversity.

In zones of spring wheat production in Russia and Kazakhstan, genes with high effectiveness in response to leaf rust (*Lr24*, *Lr28*, *Lr29*, *Lr39*, *Lr45*, *Lr47*, *Lr51*, *Lr6Agi1 Lr6Agi2* and *LrAspLrAsp*), stem rust (*Sr24*, *Sr30* and *Sr31*) and yellow rust (*Yr5*, *Yr10*, *Yr15* and *Yr24*) were described [13,14,15]. The *Lr9* and *Lr19* genes have lost their effectiveness as single genes in the regions where the cultivars having them are widely grown (Volga, Urals, Siberia and northern Kazakhstan). However, the combination of *Lr9* and *Lr19* genes and their combination with *Lr26* appear to be effective [16]. Adult plant resistance genes *Lr34*, *Lr37*, *Sr57* and *Yr18* and seedling race-specific resistance genes *Lr21*, *Sr38* and *Yr17* are moderately effective in Russia and Kazakhstan [14,15,16]. Molecular markers have been created for most of these genes, which allows for the successful identification of these genes [17].

Successful wheat breeding for rust resistance requires continuous monitoring of the efficacy of resistance genes and the evaluation of the effect of new wheat cultivars on pathogen virulence. The diversity of *P. triticina* populations in the West Asian part of Russia and northern Kazakhstan in terms of virulence and microsatellite loci has previously been studied [18]. Leaf rust samples were collected from wheat accessions tested within KASIB in 2016 at different points in Russia and Kazakhstan. A high similarity of leaf rust populations in the West Asian part of Russia and northern Kazakhstan was found for both markers. This indicates the existence of a single population of *P. triticina* in this area. The frequent emergence and rapid spread of more virulent and aggressive rust pathotypes pose a serious threat to wheat in these countries as well as to neighboring countries. The application of the strategy of mosaic cultivar placement, taking into account the optimal areas occupied by genetically similar genotypes, will stabilize the phytosanitary situation for rust in these regions. In this respect, continuous screening of new cultivars and promising breeding material for rust resistance and identification of rust resistance genes are needed. The objective of the work presented here was to evaluate seedling resistance to leaf, stem and yellow rust in new KASIB genotypes, to identify the rust resistance genes using molecular markers and multi-pathogen tests and to explore the rust resistance diversity of these genotypes.

## 2. Results

### 2.1. Identification of Rust Resistance Genes Using Molecular Markers

Molecular markers were used for the identification of 21 *Lr* genes (*Lr1*, *Lr3*, *Lr9*, *Lr10*, *Lr19*, *Lr20*, *Lr21*, *Lr24*, *Lr25*, *Lr26*, *Lr28*, *Lr29*, *Lr34*, *Lr35*, *Lr37*, *Lr41(39*), *Lr47*, *Lr51*, *LrAsp*, *Lr6Agi1* and *Lr6Agi2*), seven *Sr* genes (*Sr15*, *Sr24*, *Sr25*, *Sr31*, *Sr38*, *Sr39* and *Sr57*), seven *Yr* genes (*Yr5*, *Yr9*, *Yr10*, *Yr15*, *Yr17*, *Yr18* and *Yr24*) and 1AL.1RS translocation. The 1RS translocation carries the stem rust resistance gene *SrR*, but no known leaf and stripe resistance genes [19].

Leaf rust resistance genes *Lr1*, *Lr3*, *Lr9*, *Lr10*, *Lr19*, *Lr20*, *Lr24*, *Lr26*, *LrAsp*, *Lr6Agi1*, *Lr6Agi2*; stem rust resistance genes *Sr15*, *Sr24*, *Sr25*, *Sr31*, *Sr57*; yellow rust resistance genes *Yr9* and *Yr18* genes; and the 1AL.1RS translocation were detected in KASIB accessions (Table 1, Figure S1). The most frequent rust genes identified alone, or in combination, were *Lr3* (17 accessions); *Lr26*, *Sr31* and *Yr9* (13 accessions); *Lr10* (10 accessions); *Lr1* (9 accessions); and *Lr19* and *Sr25* (7 accessions). The *Lr9*, *Lr21* and *LrAsp* genes and the 1AL.1RS translocation were identified in two accessions, and the *Lr20*, *Lr24*, *Lr34*, *Lr6Agi1*, *Lr6Agi2*, *Sr15*, *Sr24* and *Sr57* genes in one of these. No identified rust resistance genes were detected in eight genotypes.

### 2.2. Seedling Resistance Test

Leaf rust: Seedling infection type data for 40 promising spring wheat accessions inoculated with four *P. triticina* pathotypes are given in Table 1. Three Kazakh (16%) and thirteen Russian (62%) accessions were resistant to all pathotypes. According to molecular assessment, 10 of these resistant genotypes have highly effective resistant genes *Lr6Agi1*, *Lr6Agi2* and *LrAsp* and the effective combination of *Lr19* (or *Lr9*) with *Lr26*.

The leaf rust isolates differed in their virulence to the *Lr2a*, *Lr2b*, *Lr2c*, *Lr9*, *Lr15*, *Lr19* and *Lr26* genes. A multi-pathogen test revealed the *Lr26* gene in seven wheat accessions (Lutescens 54 190/09, PCIb12I453, 249-A-25, L-6/SM, KS 29/17y, Kasibovskaya 2 and Zagadka). These genotypes were resistant (reaction type R) to PtK1 and PtK2 isolates avirulent to *Lr26* and susceptible to virulent PtK3 and PtK4 isolates. Gene *Lr9* was postulated in Lutescens 34-16 and gene *Lr19* in Lutescens 1535, which were susceptible to PtK2 and PtK1 isolates, respectively. Overall, the results of the phytopathological study were in agreement with the molecular marker data.

The multi-pathogen tests revealed the absence of the *Lr2a*, *Lr2b*, *Lr2c* and *Lr15* genes in the studied wheat collection. *P. triticina* isolate PtK4 differed from other ones for avirulence to the *Lr2a*, *Lr2b*, *Lr2c* and *Lr15* genes. Accessions resistant to PtK4 isolate and susceptible to the other isolates tested were not detected.

**Figure 1:**
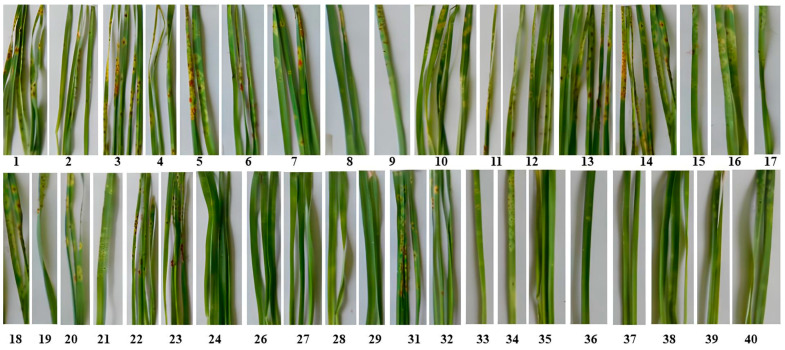
Response of KASIB accessions to race TTKTF of *Puccinia graminis* at the seedling stage. 1–40 wheat accessions according to Table 1.

Stem rust: High (R) and moderate (MR) resistance types to stem rust were determined for 4 (21%) Kazakh and 17 (81%) Russian accessions (Table 1). Figure 1 illustrates the rust responses to PgK1 isolate (race TTKTF). Thirteen (62%) resistant genotypes have *Sr25* and *Sr31* as single genes or in combination according to the molecular study.

*P. graminis* isolates differed in virulence to the *Sr9d*, *Sr9g*, *Sr17* and *Sr30* genes. The multi-pathogen tests revealed the absence of these genes in the studied collection because reaction types in response to both *P. graminis* isolates (PgK1 and PgK2) were mostly similar (Table 1).

Yellow rust: The number of accessions resistant to yellow rust was significantly lower than that for stem rust and leaf rust. Only four Russian accessions had resistant reactions to all *P. striiformis* isolates (lines L-407/ChT, L-235/PT, L447 and Lutescens 1485). Using molecular markers, highly effective resistant genes *Yr5*, *Yr10*, *Yr15* and *Yr24* were not identified in these genotypes. Gene *Yr18* was postulated for line L-407/ChT, gene *Lr9* for line L447, and 1AL.1RS translocation with no known stripe rust resistance genes for line L-235/PT, but these genes on their own are not effective against yellow rust in Russia and Kazakhstan.

Yellow rust isolates differed in virulence to *Yr1*, *Yr4*, *Yr7*, *Yr27*, *YrSD* and *YrND* genes. Multi-pathogen testing did not reveal any of these *Yr* genes in the wheat accessions tested.

Overall, in the seedling resistance study, three lines resistant to all rust species (L447, L-407/ChT and Lutescens 1485) were identified. Genes *Lr3*, *Lr10*, *Lr26*, *Lr6Agi1*, *Sr31* and *Yr9* were postulated as present in line L447; genes *Lr10*, *Lr34*, *Sr57* and *Yr18* in line L-407/ChT; and the *Lr6Agi2* gene in Lutescens 1485. Genes *Lr6Agi1* and *Lr6Agi2* can provide effective resistance to leaf, stem and yellow rust pathogens in lines L447 and Lutescens 1485. Rust resistance genes identified for line L-407/ChT could not provide a high degree of protection. Accordingly, it may have other genes whose markers were not used in this work. This line has a complex pedigree (Lutescens 148-97-16//FRTL/2*PIFED/5/Seri*3//RL6010/4*YR/3/Pastor/4) and was developed using CIMMYT material.

Nine accessions were resistant to leaf and stem rust. According to the molecular study, six of these genotypes (1616ae14, L373, Lutescens 1510, 205/12-5, 242/13-10 and 74/16-1) have the combination of the *Lr19/Sr25* and *Lr26/Sr31* genes. Genes *Sr25* and *Sr31* are highly effective against stem rust in both countries. Genes *Lr26* and *Lr19* have lost effectiveness when deployed alone, but they are still effective in combination. Gene *LrAsp* was postulated in cv. Pamyati Tynina and Erythrospermum 26464. This gene provides a high degree of resistance to leaf rust and moderate resistance to stem rust. None of the identified genes were found in cv. Kudesnitsa. This cultivar was bred using synthetic hexaploid wheats developed by CIMMYT (Lutescens 30-94/3/*T.dicoccon* pi94625/*Ae.squarrosa* (372)//3*Pastor) and probably has new *Lr* and *Sr* genes.

### 2.3. Rust Assessment in the Field

The accessions were field-tested in 2023 in the South Urals agroclimatic zone of Russia in experimental fields of Chelyabinsk Scientific Research Institute of Agriculture (Chebarkul’sk district 54.93° N, 60.74 E) under natural leaf rust infection. The leaf rust development varied in susceptible cultivars (St) from 10 to 50% (Table 2). Among the new KASIB accessions, the highest disease severity (40–50%) was detected in lines with ineffective genes *Lr1*, *Lr3* and *Lr10* as single genes or in combination (line 155-A-1 (*Lr10*), line 218/10 (*Lr1* + *Lr3*), and lines 98-A-2 and 249-A-25 (*Lr3 + Lr10*)). With the exception of 249-A-25, all these lines were highly susceptible at the seedling stage. Lines with *Lr26* as a single gene (Lutescens 54–190/09) or in combination with *Lr3* (L-6/SM) or *Lr1* (KS 39/08-7) had disease severity from 1 to 20%. Disease severity in Lutescens 1535 with *Lr19* and *Lr3* genes fluctuated from 1 to 5% with a moderate susceptible reaction type (IT 2–3 and X). Lines with the *Lr9* gene and the 1Al.1RS translocation had a disease severity of 1%. At the same time, cv. Tertsia with the gene *Lr9* as a single gene was affected more (40–50%). Symptoms of leaf rust on wheat accessions with the *Lr9* and *Lr19* genes confirm the presence of isolates with virulence against the *Lr9* and *Lr19* genes in the regional *P. triticina* population.

Most KASB-24 accessions that were resistant to leaf rust at the seedling stage were free from rust infection in the field. Only one line, L1353 with the *Lr1*, *Lr3* and *Lr10* genes, was slightly affected in the field (1%) with the moderate resistance type (IT 1–2) (Table 2).

The development of stem rust in 2023 did not allow an evaluation of the resistance of the accessions tested. Only one line, PCIb12II189, was strongly affected (30%). But this line was moderately resistant to two *P. graminis* isolates at the seedling stage. Limited development of stem rust on single plants (disease severity 5%) was found in line L-235/PT, which was segregated for resistance in the seedling test. Of the four susceptible standards, stem rust was only detected in cv. Pamyati Azieva (1%).

The agronomic performance of 1000-grain weight and grain yield of spring wheat germplasm in the South Urals are given in Table 2. Substantial yield variation was observed between accessions (from 5.01 to 2.06 t/ha). The highest grain yields were found for susceptible lines 98-A-2 and 249-A-25 with the *Lr3* and *Lr10* genes (5.01 and 4.55 t/ha). Closer to them in yield was the leaf- and stem-rust-resistant cv. Pamyati Tynina with the highly effective resistance gene *LrASp* (4.39 t/ha). However, line Erythrospermum 26,464 with a similar gene had a lower yield (3.48 t/ha). The grain yield for resistant lines with a combination of the *Lr19* and *Lr26* genes varied from 4.05 to 3.24 t/ha. Resistant lines with genes *Lr6Agi1* and *Lr6Agi2* had corresponding grain yields of 3.34 and 3.58 t/ha. Thus, substantial variation was seen for resistant accessions with alien rust genes.

## 3. Discussion

This study has screened the 40 new and promising spring wheat genotypes developed by seven Kazakh and eight Russian breeding programs for leaf, stem and yellow rust resistance. Overall, 40% of the germplasm tested had resistance to *P. triticina*, 52% to *P. graminis* and 10% to *P. striiformis*. Of these, three accessions (7.5%) were resistant to all rust species, and nine accessions (22%) to leaf and stem rust. The number of resistant genotypes was higher in the Russian collection.

The use of molecular markers enables the flow and the build-up of resistance in the wheat germplasm to be monitored and the determination of the underlying genetic diversity. In the material studied, leaf rust resistance genes *Lr1*, *Lr3*, *Lr9*, *Lr10*, *Lr19*, *Lr20*, *Lr21*, *Lr24*, *Lr26*, *Lr34*, *Lr6Agi1*, *Lr2Agi2* and *LrAsp* alone or in various combinations were postulated. Of these genes, only *Lr24*, *Lr6Agi1*, *Lr6Agi2* and *LrAsp* are highly effective against leaf rust as single genes in spring wheat growing areas in Russia and Kazakhstan. Highly effective seedling resistance genes *Lr25*, *Lr28*, *Lr29*, *Lr39(41)*, *Lr47* and *Lr51* and the moderately effective adult plant resistance gene *Lr37* were not detected in the new KASIB collection despite the fact that donors of these genes have been used in Russian and Kazakh spring wheat breeding [20].

The *Lr24* gene had low distribution in Russian and Kazakh commercially grown spring wheat cultivars, but it is reported as widespread in wheat genotypes in the USA, Australia and Western Europe [21]. The *Lr24* gene is always completely associated with *Sr24*, which is highly effective against stem rust in Russia and Kazakhstan [15]. In the present study, the *Lr24* gene was detected in only one line, but it was segregated for resistance to leaf and stem rust. In previous studies of KASIB germplasm [2,11], the *Lr24* gene was detected in Kazakh cv. Aina and Russian cvs. Lider 80 and Niva 55. Cv. Aina was included in the Kazakh State Breeding Register in 2018 [22]; cvs. Lider 80 and Niva 55 were included in the Russian State Register in 2020 and 2022, respectively [23].

Alien genes *LrAsp*, *Lr6Agi1* and *Lr6Agi2* were not included in the Catalogue of Gene Symbols for Wheat [24]. They were used in breeding programs only in Russia. Gene *LrAsp* was transferred from *Aegilops speltoides.* The donor of *LrAsp* gene is a cuckoo-type line developed in N.I. Vavilov All-Russian Institute of Plant Genetic Resources (Saint Petersburg). This line came from crosses and backcrosses with bread wheat of the complex resistant amphidiploid *Triticum dicoccum* × *Ae. speltoides* and is highly resistant to leaf rust and moderately resistant to stem rust. The Gc-gene expression leads to the elimination of gametes having the recessive gc allele in the heterozygous sporophyte tissues [25]. The first commercial cultivar Chelyaba 75 with gene *LrAsp* was developed by the Chelyabinsk Research Institute of Agriculture in 2012 [25]. Adonina et al. [26], using molecular cytology analysis by C-banding and fluorescence in situ hybridization, revealed that cv. Chelyaba 75 has a 2DS.2SL translocation from *Ae. speltoides*. None of the five catalogued *Lr* genes introduced from *Ae. speltoides* are localized in this chromosome (*Lr28* in 4AL chromosome, *Lr35* in 6BL, *Lr47* in 7AS, *Lr51* in 1BL and *Lr66* in 3AS) [21,27,28,29,30,31,32].

In 2023, the new spring wheat cv. Odintcovskaya with *LrAsp* genes was included in the Russian State Register and recommended for commercial production [23]. In 2024, the first winter wheat cultivar SPbSU 300 with gene *LrAsp* was submitted for Russian State variety testing. This cultivar was developed by the National Grain Centre and named after P.P. Lukyanenko (Krasnodar). In the present study, two new accessions with *LrAsp* genes were detected, and they were highly resistant to leaf and moderately resistant to stem rust. One of them, Pamyati Tyunina, was also submitted for State variety testing.

Two groups of spring wheat cultivars that have the *Th. intermedium* (6Agi1 and 6Agi2) chromosome substitution are widely cultivated in Russia. Cvs. Belyanka, Favorit, Voevoda and Lebiedushka developed in the Federal Agrarian Scientific Centre of the South-East have substitution 6Agi1. Commercially grown cvs. Tulaikovskaya 5, Tulaikovskaya 10 and Tulaikovskaya 100 with the 6Agi2 substitution were produced in the N.M. Tulaikov Research Institute of Agriculture. Resistance genes located in 6Agi1 and 6Agi2 chromosomes and conferring resistance to rust are not identical [33]. In 2018–2020, Ivanova et al. [6] evaluated fungal disease resistance in bread wheat hybrid lines with chromosome 6Agi2 in Western Siberia. They found that chromosome 6Agi2 enables plants to retain immunity to the West Siberian population of leaf rust and to dominant races of stem rust. It also provides medium-resistant and medium-susceptible types of response to yellow rust [6]. In the present study, two lines carrying the *Th. intermedium* (6Agi) chromosome substitution were postulated as present. Line L447 had the *Lr6Agi1* gene, and Lutescens 1485 had the *Lr6Agi2* gene. Both these lines were highly resistant to three rust species.

Widespread production of cultivars with the *Lr9* gene in the Russian Ural and Siberia regions and northern Kazakhstan in 1995–2010 led to the pathogen overcoming their resistance in these regions, but this gene is still effective in European Russian regions. The *Lr19* gene lost effectiveness in 1990 in Volga regions and later in other Russian and Kazakh regions. Effective combinations of these genes with other effective and partially effective genes can significantly increase field resistance. A combination of gene *Lr9* (or *Lr19*) and gene *Lr26* is effective against Russian and Kazakh *P. triticina* populations. Isolates virulent to *Lr9*, *Lr19* and *Lr26*, as single genes, have a high distribution in local populations. Previously, we did not know of isolates virulent to a combination of these genes (*Lr9* + *Lr26*, *Lr19* + *Lr26* and *Lr9* + *Lr19*), and this has enabled the continued effectiveness of these combinations [13]. However, in pathogen populations, there is widespread virulence to *Lr19* (or *Lr9*) along with *Lr1*, *Lr2a*, *Lr2b*, *Lr2c*, *Lr3a*, *Lr3bg*, *Lr3ka*, *Lr10*, *Lr14a*, *Lr14b*, *Lr15*, *Lr17*, *Lr18*, *Lr20* and *Lr30* [13,18].

Commercial spring wheat cultivars with the *Lr19* and *Lr26* genes (Omskaya 37, Omskaya 38, Omskaya 44) and the *Lr9* and *Lr26* genes (Chelyabinka, Silach) have been grown widely in West Siberia and the Urals [23] since 2010 and remain resistant to leaf rust. In the present study, six lines from three geographically separated Russian breeding centers (Samara, Saratov and Omsk) with the combination of the *Lr19* and *Lr26* genes were postulated. Thus, progress has now been made in developing wheat cultivars with a combination of these genes. The *Sr31* and *Sr25* genes, tightly linked with the *Lr26* and *Lr19* genes, are highly effective in response to stem rust in both countries. All lines with these genes were mostly susceptible to yellow rust at the seedling stage.

The *Lr21* gene was introduced to common wheat from *Ae. tauschii* [21] and until 2020 was not postulated as present in Russian and Kazakh commercial cultivars. Initially, it was tested in spring wheat cv. Silantiy studied in KASIB in 2019–2020 and included in the Russian State Register in 2022. In the pedigree of this cultivar, there is synthetic hexaploid wheat with genetic material of *Ae. tauschii* from SIMMYT wheat programs. In the present study, two lines with the *Lr21* gene were detected (201m/22 and L373). Line L373 developed by the Federal Agrarian Scientific Centre of the South-East (Russia) had a complex of rust genes, *Lr3*, *Lr10*, *Lr19*, *Lr21*, *Lr26*, *Sr25*, *Sr31* and *Yr9*, and was resistant to all isolates of *P. triticina* and *P. graminis* and to four of five isolates of *P. striiformis*. The source of the *Lr21* gene for this line is also the synthetic amphidiploid from SIMMYT (L505/3/Croc/*Ae.squar*(205)//Weaver/4/Л505/5/S68). At the same time, the *Lr21* gene was not detected in cv. Kudesnitsa, which was developed using another accession of *Ae. tauschii* amphidiploid (Lutescens 30–94/3/*T.dicoccon* pi94625/*Ae.squar*(372)//3*Pastor). Kazakh line 201m/22 was developed using cvs. Stepnaya 18 and Tulaikovskaya 1 without the *Lr21* gene. The used *P. triticina* isolates had moderately susceptible infection types on the Thatcher line with the *Lr21* gene. Thus, the amplification of the marker in this strain may have been a false positive.

The *Yr5*, *Yr10*, *Yr15* and *Yr24* genes, which are highly effective against yellow rust in both countries, were not postulated as present in three accessions resistant to all used *P. striiformis* isolates. This suggests that they have new genes or efficient combinations of other genes.

The gene *Yr9* has a high distribution in the studied KASIB-24 wheat collection. Most accessions with *Yr9* were susceptible to yellow rust at the seedling stage. The *Yr9* gene lost its effectiveness around the world, including in Russia and Kazakhstan [14,34,35]. Nevertheless, it can be used in combination with other seedling resistance or non-race-specific or partial *Yr* genes. This technique is becoming popular and most important for developing and improving the output of breeding to obtain broad-spectrum resistance capabilities.

All effective rust resistance genes in the new KASIB-24 germplasm are alien and have been transferred from wheatgrass, *Aegilops* and wild relatives. Chromosomal translocations in wheat derived from alien species are a valuable source of genetic diversity that have provided increases in resistance to various diseases and improved tolerance to abiotic stresses in wheat [36]. The chromosomal translocation involving 1RS rye (*Secale cereale*) and 1BL wheat (*Triticum aestivum*) has been one of the most widely used sources of alien genetic material in wheat cultivars around the world. It is also widely present in previous [2,11,12] and new KASIB collections. The short arm of the 1R chromosome carries the *Lr26*, *Sr31*, *Yr9* and *Pm8* genes and is associated with increased yield potential across a wide range of environments [37]. The second representation in the KASIB-24 collection was shown by accessions with *Lr19/Sr25* genes transferred to wheat from *Th. ponticum* [2,11,12]. It has also been shown that this translocation was associated with increases in yield, final biomass and grain number [38]. In the present study, the grain yield of accessions with the 1BL.1RS and *Lr19/Sr25* translocations, alone or in combination, varied greatly. A similar result was obtained for accessions with the *LrAsp* gene.

The moderately effective adult plant resistance genes *Lr37*, *Sr38* and *Yr17* were not postulated in this study. However, they have been identified in the previously studied KASIB collections [2,10,11,12]. These resistance genes have been incorporated into wheat cultivars in Northern Europe since the mid-1970s and have been widely used in CIMMYT breeding programs. The CIMMYT genotypes with the *Lr37*, *Sr38* and *Yr17* genes were the sources of these genes in previously studied KASIB accessions.

The locus with adult plant resistance genes *Lr34*, *Sr67*, *Yr18* and *Pm38* confers quantitative or partial resistance against multiple biotrophic pathogens. This is due to the pleiotropic effect of there being only one resistance gene. A similar effect was shown for loci with the *Lr46*, *Sr58*, *Pm39*, *Yr29* genes and *Lr67*, *Sr55*, *Yr46*, *Pm46* genes [18], but they were not used for KASIB breeding. In the presented molecular analysis, the *Lr34*, *Sr67*, *Yr18* genes were detected only for one line. Remarkably, the role of *Lr34* in leaf rust genetic protection in the KASIB germplasm was relatively weak in previous studies [2,10,11,12]. But these genes are most prevalent in Russian commercial winter wheat germplasm [16].

Multi-pathogen tests with an array of pathotypes differing in virulence genes are also widely used to determine genetic diversity for rust resistance. In this study, only *Lr9*, *Lr19* and *Lr26* as single resistance genes were determined in some accessions. Stem and yellow rust resistance genes have not been postulated. The diagnostic ability of this method was lower as most wheat accessions with a combination of resistance genes and no virulent isolates were identified in Russia and Kazakhstan. Molecular markers can detect target genes in germplasm collections in the absence of appropriate pathogen isolates.

Studies of KASIB genotypes in response to leaf rust using both methods have been conducted since 2015. In 2016–2019, 120 cultivars and breeding lines from the KASIB study in 2000–2016 with differing degrees of resistance to leaf rust were analyzed [2]. It was shown that the most frequent *Lr* genes identified in the KASIB germplasm alone or in combination were *Lr1*, *Lr9*, *Lr10*, *Lr17*, *Lr19*, *Lr26* and *Lr34.* The *Lr14a*, *Lr24*, *Lr37*, *Lr39* and *LrAsp* genes were identified in up to two genotypes each. An increasing diversity of *Lr* genes was found in the tested KASIB spring wheat accessions from 2019 to 2022. In addition to the above genes, the *Lr3*, *Lr20*, *Lr21*, *Lr6Agi2* genes were identified [10,11,12]. The present study analyzed the resistance of new KASIB accessions to three rust species, and a large number of genotypes resistant to leaf and stem rust separately and in combination were identified. Most of these had either highly or partially effective single resistance genes or combinations of resistance genes that make it possible to maintain resistance levels over a long period of time. At the same time, global climate change and changes in the composition of pathogens in wheat growing areas require greater emphasis on advanced breeding, including breeding for resistance to yellow rust.

## 4. Materials and Methods

### 4.1. Plant Material

The study included 40 advanced spring wheat genotypes developed in seven Kazakhstan and eight Russian Wheat Breeding Centers and supplied for testing in KASIB trials in 2023–2024 (Table 3).

### 4.2. Identification of Lr, Sr and Yr Genes Using Molecular Markers

Molecular markers used for identification of *Lr*, *Sr* and *Yr* genes are presented in Table 4. DNA was extracted according to Dorokhov and Klocke [39]. PCRs were performed using a thermocycler (C1000, BioRad, Hercules, CA, USA). The PCR mixture (20 mL) contained 50–150 ng of genomic DNA, 2 units of Taq DNA polymerase, 1× PCR buffer, 2.5 mM of MgCl2, 100 µM of each dNTP and 10 pM of each primer. The recommended PCR protocol (Table 4) was used in amplifications. PCR products were separated on 1.5 to 3% agarose gels (depending on gene product size) and visualized under UV light using a digital gel imaging system (GelDocGo, BioRad, Hercules, CA, USA).

### 4.3. Seedling Tests

Leaf rust infection at the seedling stage was evaluated for four *P. triticina* isolates (*Pt*) with different virulence–avirulence combinations, stem rust infection for two *P. graminis* isolates (Pg), yellow rust for five *P. striiformis* isolates (Pst). Virulence–avirulence profiles of rust isolates at the seedling stage are presented in Table 5. Near isogenic Thatcher lines with *Lr* genes 1, 2a, 2b, 2c, 3a, 3bg, 3ka, 9, 10, 14a, 14b, 15, 16, 17, 18, 19, 20, 24, 26, 28, 29, 30, 47 and 51 were used for virulence characterization of *P. triticina* isolates. Near isogenic Marques lines with *Sr* genes 5, 6, 7b, 8a, 9a, 9b, 9g, 9e, 9d, 10, 11, 17, 21, 24, 24 + 31, 24 + 36, 25, 30, 31 were likewise used for *P. graminis* isolates, and near-isogenic Avocet lines with *Yr* genes 1, 5a, 6, 7, 8, 9 10, 15, 17, 24, 27 and *5b* (*YrSp* = Spalding prolific) and differentials Heines VII (*Yr2*), Vilmorin 23 (*Yr3*), Hybrid 46 (*Yr4*), Nord Desprez (*YrND*), Strubes Dickkopf (*YrSD*) were used for *P. striiformis* isolates.

Based on the North American system of nomenclature [58] and additional sets describing the variation in Russia [13], *P. triticina* isolate PtK1 belonged to the TLT/TR race, isolate PtK2 to the TGT/TT race, isolate PtK3 to the THT/TR race and isolate PtK4 to the MHT/KH race. Avirulence/virulence was determined with the following differential sets: group I: *Lr1*, *Lr2a*, *Lr2c* and *Lr3a;* group II: *Lr9*, *Lr16*, *Lr24* and *Lr26*; group III: *Lr3ka*, *Lr11*, *Lr17* and *Lr30*; group IV: *Lr2b*, *Lr3bg*, *Lr14a* and *Lr14b*; and group V: *Lr15*, *Lr18*, *Lr19* and *Lr20.* The designation of races for stem rust was performed using the following international differential sets: group I: *Sr5*, *Sr21*, *Sr9e* and *Sr7b*: group II: *Sr9a11*, *Sr6*, *Sr8a* and *Sr9g*; group III: *Sr36*, *Sr9b*, *Sr30* and *Sr17*; group IV: *Sr9a*, *Sr9d*, *Sr10* and *SrTmp*; and group IV: *Sr24*, *Sr31*, *Sr38* and *SrMcN* [59]. Accordingly, isolate PgK1 was designated as race TTKTF and isolate PgK1 as race TSGPF, as no international race designation system has been proposed for the yellow rust pathogen.

For rust resistance assessments performed at the seedling stage, 8–10-day-old plants (with the primary leaves fully emerged) were used for inoculation by *P. triticina* and *P. graminis*, and 12–14-day-old plants (with emerged second leaf) for *P. striiformis*. Three to ten seeds of each genotype were planted in 10 cm diameter plastic pots in a disease-free area. Urediniospores of a single isolate were suspended in non-phytotoxic mineral oil Novec 7100 in a glass tube and connected to the airbrush spray gun. The plants inoculated by leaf and stem rust were incubated in a dark dew chamber at 20 °C for 24 h and then transferred to a growth chamber with a 16:8 h L/D photoperiod at a constant 20 °C. The plants inoculated by yellow rust were incubated in a dark dew chamber at 10 °C for 24 h and then transferred to a growth chamber (Environmental Test Chamber MLR-352H, Sanyo Electric Co., Ltd., Osaka, Japan) with 16:8 h L/D photoperiod at 16 and 10 °C, respectively [16].

Seedlings were assessed for their infection types according to Mains and Jackson [60] for leaf rust (10–12 d after inoculation), Stakman and Levin [61] for stem rust (12–14 d after inoculation), Gassner and Straib [62] for yellow rust (16–18 d after inoculation).

### 4.4. Disease Assessment in the Field

The accessions were field-tested in 2023 in the South Urals agroclimatic zone of Russia in experimental fields of Chelyabinsk Scientific Research Institute of Agriculture (Chebarkul’sk district, 54.93° N, 60.74° E). Spring wheat trials had 3-m^2^ plots each with three replicates. Rust reactions were evaluated under natural infection. Leaf and stem rust resistance of spring wheat accessions was evaluated using the modified Cobb scale [63]. The scoring was based both on disease severity (proportion of leaf area infected) and on the plant response to infection (reaction type). Plant responses were recorded as resistant (R), moderately resistant (MR), moderately susceptible (MS), and susceptible (S) reactions [64]. Cvs. Pamyati Azieva (*Lr10*), Tertsiya (*Lr9*), Omskaya 35 (*Lr10*) and Saratovskaya 29 (*Lr10*) were used as the susceptible controls (standards, St).

Weather conditions in 2023 were not favorable for rust development. Precipitation in the second ten days of May, given the good soil moisture reserves prior to sowing, favored early emergence despite the elevated air temperatures. Rainfall in June was in line with the annual average. July was unusually hot and dry: air temperature rose to 35–40 °C, soil temperature to 55–57 °C, rainfall was only 13.3 mm and there was a hydrothermal coefficient of 0.2. From 7 August, when the cultivars were close to ripening, the rain became prolonged. In the first and third 10 days of August, 115 and 101 mm of rain fell, giving a total of 218 mm for August, and there was a GTC of 4.2 with a norm of 1.2.

The first symptoms of leaf rust were noted in the phase of milk-wax ripeness, and stem rust in the phase of wax ripeness. The leaf rust development on the susceptible cultivars varied strongly (cv. Tertciya, 50%; cv. Saratovskaya, 40%; cv. Omskaya 35, 20%; and cv. Pamyati Azieva, 10%). The highest stem rust development (30%) was observed in line PCI b12I I189.

Additionally, the agronomic performance of 1000-grain weight and grain yield of spring wheat germplasm in Chelyabinsk (54°93 N, 60°74 E) in 2023 was determined. All entries were harvested, and yield components (1000-grain weight and yield) were evaluated following the methods of Pietragalla and Pask [65]. Statistical analysis was limited to ANOVA for yield components.

## 5. Conclusions

The new spring bread wheat collection from Kazakhstan-Siberia Network for Spring Wheat Improvement (KASIB-24) including 40 promising cultivars and breeding lines of Russian and Kazakh breeding (19 and 21, respectively) was characterized for resistance to three rust species. As a result, 16 breeding accessions with seedling resistance to leaf rust, 21 with resistance to stem rust and 4 with resistance to yellow rust were identified. Three breeding accessions were resistant to all rust species, and nine to *P. triticina* and *P. graminis*. The number of resistant genotypes was higher in the Russian collection. All wheat accessions highly resistant to leaf rust at the seedling stage were also resistant in the field in the South Urals agroclimatic zone of Russia in 2023.

High levels of diversity for rusts were found among accessions. Molecular analysis showed the presence of cataloged resistance genes *Lr1*, *Lr3a*, *Lr9*, *Lr10*, *Lr19*, *Lr20*, *Lr24*, *Lr26*, *Sr15*, *Sr24*, *Sr25*, *Sr31*, *Sr38*, *Yr9* and *Yr18*; uncatalogued genes *Lr6Agi1* and *Lr6Agi2* from *T. intermedium* and *LrAsp* from *Ae. speltoides*; and 1AL.1RS translocation with the stem rust resistance gene *SrR* and no known leaf and yellow rust resistance genes.

The current rust resistance in KASIB spring wheat is effective, as resistance against various isolates has been introduced into promising genotypes. Breeding progress in rust resistance of KASIB genotypes can mostly be attributed to effective all-stage resistance genes alone or in effective combinations. The breeding strategies implemented for improved resistance have clearly been highly successful. Nevertheless, global climate change and changes in the composition of pathogens in wheat growing areas mean that ongoing work is needed to advance breeding, especially for resistance to yellow rust.

The diverse spring wheat accessions with rust resistance found in our study can be used in breeding programs for developing new wheat cultivars. The introduction of improved cultivars will prevent disease yield losses in Russia and in Kazakhstan, providing a direct contribution to yield increase.

## Figures and Tables

**Table 1:** Reaction of spring wheat germplasm to rusts at the seedling stage and identified rust resistance genes.

	Entry	Reaction Type to Rust Isolates at the Seedling Stage											Identified Rust Resistance Genes
		*P. triticina*				*P. graminis*		*P. striiformis*					
		PtK1	PtK2	PtK3	PtK4	PgK1	PgK2	PstK1	PstK2	PstK3	PstK4	PstK5	
1	Line 201m/22	S	S	S	S	S	S	S	S	S	S	S	*Lr3 Lr10 Lr21*
2	Line 334m/22	MR	MR	MR	MR	MS	MS	R	MR	R	S	MS	-
3	Line 337m/22	MR	MR	S	MR	S	S	S	S	S	S	S	*Lr10*
4	Line 55/08	S	S	S	S	MS-S	S	S	S	S	S	S	*Lr3*
5	Line 143/09	S	S	S	S	S	S	S	S	S	S	S	*-*
6	Line 42/93-09-1	R	R	MR	R	S	S	S	S	S	S	MS	*Lr3*
7	Line 1205-09-8	S	S	S	S	MS-S	S	R	MR	S	S	MR	*-*
8	Lutescens 54 190/09	MR	MR	MS	S	MR	MR	S	S	S	S	S	*Lr26 Sr31 Yr9*
9	Lutescens 20 161/08	S	S	S	S	MS-S	S	R	S	S	S	S	*-*
10	Kudesnitsa	R	R	R	R	MR	MR	S	S	S	S	S	*-*
11	Lutescence 2216	S	S	S	S	S	S	S	S	S	S	S	*Lr3*
12	Lutescence 2222	S	S	S	S	MS-S	MS	S	S	S	S	S	*-*
13	Saru Akca 27	S	S	S	S	S	S	S	S	S	S	S	*Lr1 Lr3*
14	Line 218/10	S	S	S	S	S	S	S	S	S	S	S	*Lr1 Lr3*
15	PCIb12I453	R	R	S	S	MR	MR	S	S	S	S	S	*Lr3 Lr20 Lr26 Sr15 Sr31 Yr9*
16	PCIb12I I189	S	S	S	S	MR	MR	S	S	S	S	S	*Lr1 Lr3*
17	Line 98-A-2	S	S	S	S	S	MS	S	S	S	S	S	*Lr3 Lr10*
18	Line 155-A-1	S	S	S	S	S	S	S	S	S	S	S	*Lr10*
19	Line 249-A-25	R	R	S	S	MS	MS	S	S	S	S	S	*Lr3 Lr10*
20	L-407/ChT	R	R	R	R	MR	MR	R	R	R	R	R	*Lr10 Lr34 Sr57 Yr18*
21	L-6/SM	R	R	S	S	R-MR	R-MR	S	R	R	S	S	*Lr3 Lr26 Sr31 Yr9*
22	L-235/PT	R,S	R	MR	R, S	MR S	MR, S	R	R	MR	R	R	*Lr9 Lr24 Sr24 1AL/1RS*
23	KS 39/08-7	S	S	S	S	S	S	S	S	S	S	S	*-*
24	KS 29/17y	R	R	S	S	R-MR	R-MR	S	S	S	S	S	*Lr1 Lr26 Yr31 Yr9*
25	Lutescens 1485	R	R	R	R	MR	MR	R	R	R	MR	R	*Lr6Agi2*
26	Lutescens 1510	R	R	R	R	R	R	MR	MR	S	S	MS	*Lr19 Lr26 Sr25 Sr31 Yr9*
27	Lutescens 1535	R	S	R	R	R	R	S	MS	S	S	S	*Lr3 Lr19 Sr25*
28	Line 1616ae14	R	R	R	R	R	R	S	R	R	S	S	*Lr19 Lr26 Yr9 Sr25 Sr31*
29	L373	R	R	R	R	R	R	R	S	MR	R	R	*Lr3 Lr10 Lr19 Lr21 Lr26 Sr25 Sr31 Yr9*
30	L447	R	R	R	R	R	R	R	R	R	R	R	*Lr3 Lr10 Lr26 Lr6Agi1 Sr31 Yr9*
31	L2203	S	S	S	S	S	S	S	S	S	S	S	*-*
32	L1353	R	R	MR	R	MS	MS	S	S	S	S	S	*Lr1 Lr3 Lr10*
33	Kasibovskaya 2	R	R	S	S	R-MR	R-MR	MR	MR	S	S	MS	*Lr1 Lr26 Sr31 Yr9*
34	Lutescens 34–16	S	R	R	R	MR	MR	S	S	S	S	S	*Lr9 AL.1RS*
35	Lutescens 205/12-5	R	R	R	R	R	R	S	S	S	S	S	*Lr1 Lr3 Lr19 Lr26 Sr25 Sr31 Yr9*
36	Lutescens 242/13-10	R	R	R	R	R	R	S	S	S	S	S	*Lr3 Lr19 Lr26 Sr25 Sr31 Yr9*
37	Lutescens 74/16-1	R	R	R	R	R	R	S	S	S	S	S	*Lr19 Lr26 Sr25 Sr31 Yr9*
38	Pamyaty Tynina	R	R	R	R	R-MR	R-MR	S	S	S	S	S	*Lr3 LrAsp*
39	Zagadka	R	R	S	S	MR	MR	S	S	S	S	S	*Lr1 Lr10 Lr26 Sr31 Yr9*
40	Erythrospermum 26464	R	R	R	R	MR	MR	S	S	S	S	S	*Lr1 Lr3 LrAsp*

Reaction types were 0 for resistance (R) and 4 for susceptibility (S), with 1–2 for moderately resistant (MR) and 2, 3(X) and 3 for moderately susceptible (MS). Lines 1–19, Kazakh; lines 20–40, Russian accessions.

**Table 2:** Leaf rust severity of KASIB-24 accessions in the field in South Urals agroclimatic zone in 2023 and agronomic performance of 1000-grain weight and grain yield.

No.	Entry	Leaf Rust Severity and Reaction Type in the Field	1000-Grain Weight, g	Grain Yield, t/ha
1	Line 201m/22	1 MR	38.0	3.19
2	Line 334m/22	1 MS	38.5	3.88
3	Line 337m/22	1 MS	38.3	4.00
4	Line 55/08	30 S	37.1	3.83
5	Line 143/09	50 S	37.6	3.68
6	Line 42/93-09-1	5 S	36.2	4.28
7	Line 1205-09-8	10–20 S	35.1	3.93
8	Lutescens 54 190/09	1 S	33.4	3.20
9	Lutescens 20 161/08	0	35.6	3.45
10	Kudesnica	0	34.2	3.20
11	Lutescence 2216	5 S	36.6	3.61
12	Lutescence 2222	0	36.1	3.25
13	Saru Akca 27	5 MS	32.6	3.55
14	Line 218/10	50 S	42.0	2.56
15	PCIb12I453	20 S	37.6	3.24
16	PCIb12I I189	1 S	38.8	3.65
17	Line 98-A-2	50 MS	39.7	5.01
18	Line 155-A-1	40 MS	40.8	3.03
19	Line 249-A-25	50 S	38.8	4.55
20	L-407/ChT	0	34.6	2.06
21	L-6/SM	10–20 S	37.9	3.57
22	L-235/PT	0, 10 S	35.7	3.65
23	KS 39/08-7	50 S	35.2	2.47
24	KS 29/17y	1 S	44.1	3.68
25	Lutescens 1485	0	33.8	3.58
26	Lutescens 1510	0	38.2	4.02
27	Lutescens 1535	1–5 MS	41.4	4.00
28	Line 1616ae14	0	39.0	3.71
29	L373	0	36.3	4.05
30	L447	0	33.9	3.34
31	L2203	0	39.0	3.48
32	L1353	1 MR	36.8	3.42
33	Kasibovskaya 2	1 S	36.3	3.67
34	Lutescens 34-16	1 MR	38.7	2.98
35	Lutescens 205/12-5	0	38.6	3.24
36	Lutescens 242/13-10	0	37.7	3.67
37	Lutescens 74/16-1	0	33.8	3.36
38	Pamyaty Tynina	0	37.0	4.39
39	Zagadka	0	35.9	3.44
40	Erythrospermum 26464	0	34.1	3.48
St	Pamyati Azieva	10 S	36.8	3.04
St	Tertsiya	40–50 MS	36.3	3.38
St	Omskaya 35	20 S	42.7	2.73
St	Saratovskaya 29	40 S	39.5	3.22
LSD, *p* < 0.5			0.12	0.58

**Table 3:** Wheat cultivars and lines of the KASIB-24 nursery.

No.	Entry	Origin	Organization
1.	Line 201m/22	KZ: Aktyubinsk	Aktobe Agricultural Experimental Station
2.	Line 334m/22		
3.	Line 337m/22		
4.	Line 55/08	KZ: Shortandu	Scientific and Production Center of Grain Farming named after A. I. Barayev
5.	Line 143/09		
6.	Line 42/93-09-1	KZ: Pavlodar	Pavlodar Agricultural Experimental Station
7.	Line 1205-09-8		
8.	Lutescens 54 190/09	KZ: Karabalyk	Karabalyk Agricultural Experimental Station
9.	Lutescens 20 161/08		
10.	Kudesnica		
11.	Lutescence 2216	KZ: Karaganda	Karaganda Agricultural Experimental Station named after Khristenko
12.	Lutescence 2222		
13.	Saru Akca 27		
14.	Line 218/10	KZ: Akkaiyn	North Kazakhstan Agricultural Experimental Station
15.	PCIb12I 453		
16.	PCIb12I I189		
17.	Line 98-A-2	KZ: Ust-Kamenogorsk	Pilot farm of oil plants
18.	Line 155-A-1		
19.	Line 249-A-25		
20.	L-407/ChT	RU: Kurgan	Kurgan Agricultural Research Institute
21.	L-6/SM		
22.	L-235/PT		
23.	KS 39/08-7	RU: Kurgan	Research and Production Agroholding «Kurgansemena»
24.	KS 29/17y		
25.	Lutescens 1485	RU: Samara	N.M. Tulaikov Research Institute of Agriculture
26.	Lutescens 1510		
27.	Lutescens 1535		
28	Line 1616ae14		
29.	L373	RU: Saratov	Federal Agrarian Scientific Centre of the South-East
30	L447		
31.	L2203	RU: Novosibirsk	Siberian Research Institute of Plant Cultivation and Breeding—Branch of Institute of Cytology and Genetics
32.	L1353		
33.	Kasibovskaya 2	RU: Omsk	Omsk State Agrarian University
34.	Lutescens 34-16		
35.	Lutescens 205/12-5	RU: Omsk	Omsk Agrarian Centre
36.	Lutescens 242/13-10		
37	Lutescens 74/16-1		
38	Pamyaty Tynina	RU: Chelyabinsk	Chelyabinsk Research Institute of Agriculture
39	Zagadka		
40	Erythrospermum 26464		

**Table 4:** Molecular markers used for identification of *Lr*, *Sr* and *Yr* genes.

Gene	Marker	Primer Sequence	Allele Size, bp	References
*Lr1*	WR003 F/R	F: GGGACAGAGACCTTGGTGGA R: GACGATGATGATTTGCTGCTGG	760	[40]
*Lr3a*	Xmwg798	F: GGCTGTCTACATCTTCTGCA R: CAAGTGTTGAGAAGGAGAGT	365	[41]
*Lr9*	SCS5	F: TGCGCCCTTCAAAGGAAG R: TGCGCCCTTCTGAACTGTAT	550	[42]
*Lr10*	F1.2245/Lr10-6/r2	F: GTGTAATGCATGCAGGTTCC R: AGGTGTGAGTGAGTTATGTT	310	[43]
*Lr21*	Lr21F/R	F: CGCTTTTACCGAGATTGGTC R: TCTGGTATCTCACGAAGCCTT	669	[44]
*Lr25*	Lr25F20/R19	F: CCACCCAGAGTATACCAGAG R: CCACCCAGAGCTCATAGAA	1800	[45]
*Lr28*	SCS421	F: ACAAGGTAAGTCTCCAACCA R: AGTCGACCGAGATTTTAACC	570	[27]
*Lr29*	Lr29F24	F: GTGACCTCAGGCAATGCACACAGT R: GTGACCTCAGAACCGATGTCCATC	900	[45]
*Lr41(39)*	GDM35	F: CCTGCTCTGCCCTAGATACG R: ATGTGAATGTGATGCATGCA	190	[46]
*Lr47*	PS10	F: GCTGATGACCCTGACCGGT R: TCTTCATGCCCGGTCGGGT	282	[30]
*Lr51*	S30-13L/AGA7-759	F: GCATCAACAAGATATTCGTTATGACC R: TGGCTGCTCAGAAAACTGGAC	783, 422	[31]
*Lr66(Asp)*	S13-R16	F: GGTGAACGCTAAACCCAGGTAACC R: CAACCTGGGAAGATGCTGAG	695	[32]
*Yr5*	STS7/8	F: GTA CAA TTC ACC TAG AGT R GCA AGT TTT CTC CCT ATT	478	[47]
	STS9/10	F: AAA GAA TAC TTT AAT GAA R: CAA ACT TAT CAG GAT TAC	439	
*Yr10*	Xpsp3000	F: GCAGACCTGTGTCATTGGTC R: GATATAGTGGCAGCAGGATACG	220, 260	[48]
*Yr15*	Xbarc8	F: GCGGGAATCATGCATAGGA R: GCGGGGGCGAAACATACACATAAAAACA	96	[49]
*Yr24*	Barc181	F: CGCTGGAGGGGGTAAGTCATCAC R: CGCAAATCAAGAACACGGGAGAAAGAA	180	[50]
*Lr19*, *Sr25*	SCS265	F: GGCGGATAAGCAGAGCAGAG R: GGCGGATAAGTGGGTTATGG	512	[51]
*Lr20*, *Sr15*	STS638	F: ACAGCGATGAAGCAATGAAA R: GTCCAGTTGGTTGATGGAAT	542	[52]
*Lr24*, *Sr24*	Sr24≠12	F: CACCCGTGACATGCTCGTA R: AACAGGAAATGAGCAACGATGT	500	[53]
	Sr24≠50	F: CCCAGCATCGGTGAAAGAA R: ATGCGGAGCCTTCACATTTT	200	
*Lr26*, *Sr31*, *Yr9*	SCM9	F: TGACAACCCCCTTTCCCTCGT R: TCATCGACGCTAAGGAGGACCC	207(1BL.1RS) 228(1AL.1RS)	[54]
*Lr34*, *Sr57*, *Yr18*	csLV34	F: GTTGGTTAAGACTGGTGATGG R: TGCTTGCTATTGCTGAATAGT	150	[55]
*Lr35*, *Sr39*	Sr39#22r	F: AGAGAAGATAAGCAGTAAACATG R: TGCTGTCATGAGAGGAACTCTG	487	[28]
	Sr39F2/R3	F: AGAGAGAGTAGAAGAGCT R: AGAGAGAGAGCATCCACC	900	[29]
*Lr37*, *Sr38*, *Yr17*	Ventriup/LN2	F: AGGGGCTACTGACCAAGGCT R: TGCAGCTACAGCAGTATGTACACAAAA	259	[56]
*Lr_Yr6Agi2*	MF2/MR1r2	F: GATGTCG-AGGAGCATTTTC R: GTGGTAGATTACTAGAGTTCAAGTG	347	[6]
*Lr6Agi1*	j09/1 + F2 j09/1 + 4a	Not published–confidential Not published–confidential	272 269	[57] [57]

**Table 5:** Virulence–avirulence profile of *Puccinia triticina*, *Puccinia graminis* and *Puccinia striiformis* isolates.

Isolate	Origination	Virulence to Genes	Avirulence to Genes (Reaction Type)
*Puccinia triticina*			
PtK1	Chelyabinsk, 2022	*Lr*: 1, 2a, 2b, 2c, 3a, 3bg, 3ka, 9,10, 14a, 14b, 15, 17, 18, 20, 30	*Lr*: 19 (R), 16 (R), 24(R), 26(R), 28(R), 29(R), 47(R), 51(R)
PtK2	Saratov, 2021	*Lr*: 1, 2a, 2b, 2c, 3a, 3bg, 3ka, 10, 14a, 14b, 15, 16, 17, 18, 19, 20, 30	*Lr*: 9(R), 24(R), 26(R), 28(R), 29(R), 47(R), 51(R)
PtK3	Novosibirsk 2021	*Lr*: 1, 2a, 2b, 2c, 3a, 3bg, 3ka, 10, 14a, 14b, 15, 16, 17, 18, 20, 26, 30	*Lr*: 9(R), 19(R), 24(R), 28(R), 29(R), 47(R), 51(R)
PtK4	Chelyabinsk, 2022	*Lr*: 1, 3a, 3bg, 3ka, 10, 14a, 14b, 16, 17, 18, 20, 26, 30	*Lr*: 2a(R), 2b(R), 2c(MR), 9(R), 15(R), 19(R), 24(R), 28(R), 29(R), 47(R), 51(R)
*Puccinia graminis*			
PgK1	Chelyabinsk, 2022	*Sr:* 5, 6, 7b, 8a, 9a, 9b, 9g, 9e, 9d, 10, 11, 17, 21, 30, 38, Tmp, McN	*Sr*: 24(MR), 25(R), 24 + 31(R), 24 + 36(R), 31(MR), 36(R)
PgK2	Chelyabinsk, 2019	*Sr:* 5, 6, 7b, 8a, 9a, 9b, 9e, 10, 11, 21, 38, Tmp, McN	*Sr*: 9d(MR), 9g(MR), 17(MR), 24(R), 25(R), 24 + 31(R), 24 + 36(R), 30(R), 31(MR), 36(R)
*Puccinia striiformis*			
PstK1	Saratov, 2023	*Yr:* 1, 2, 3, 4, 6, 8, 9, 27, ND	*Yr:* 5(R), 7(MR), 10(R), 15(R), 17(MR), 24(R), SP(R), SD(MR)
PstK2	St Petersburg, 2022	*Yr:* 2, 3, 4, 6, 8, 9, 27	*Yr:* 1(R), 5(R), 7(MR), 10(R), 15(R), 17(MR), 24(MR), SP(R), SD(MR), ND(R)
PstK3	Krasnodar, 2022	*Yr:* 1, 2, 3, 4, 6, 7, 8, 9,	*Yr:* 5(R), 10(R), 15(R), 17(R), 24(R), 27(R), SP(R), SD(R), ND(MR)
PstK4	Dagestan, 2023	*Yr:* 2, 3, 4, 6, 7, 8, 9, 27, SD, ND	*Yr:* 1(R), 5(R), 10(R), 15(R), 17(MR), 24(R), SP(R)
PstK5	Novosibirsk, 2021	*Yr:* 1, 2, 3, 6, 8, 9, 27, SD	*Yr:* 4(MR), 5(R), 7(MR), 10(R), 15(R), 17(R), 24(R), SP(R), ND(MR)

## Data Availability

All data are provided in the manuscript.

## Supplementary Materials

The following supporting information can be downloaded at https://www.mdpi.com/article/10.3390/plants13172469/s1, Figure S1: Electrophoretogram for markers: (a) SCS265 (*Lr19* and *Sr25*), (b) SCS5 (*Lr9*), (c) F1.2245/Lr10-6/r2 (*Lr10*), (d) Sr24≠50 (*Lr24*, *Sr24*), (e) SCM9 (*Lr26*, *Sr31*, *Yr9* and 1AL.1RS), (f) STS638 (*Lr20* and Sr15), (g) MF2/MR1r2 (*Lr6Agi2*), (i) S13-R16 (*Lr66(Sp*)), (k) Lr21F/R (*Lr21*), (l) WR003 F/R (*Lr1*).
