# Supplementary material for: Genetic Diversity of Promising Spring Wheat Accessions from Russia and Kazakhstan for Rust Resistance
Source: Plants (Basel). 2024 Sep 4;13(17):2469. doi: 10.3390/plants13172469 (PMC11396946; doi:10.3390/plants13172469)
Supplement: Supplementary file 1 [file plants-13-02469-s001.zip › plants-3188895-SI.pdf]

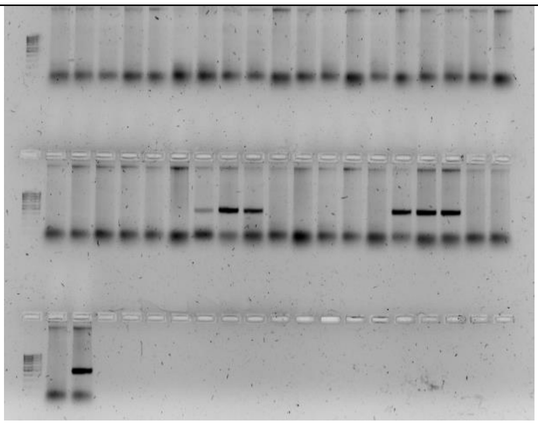

a

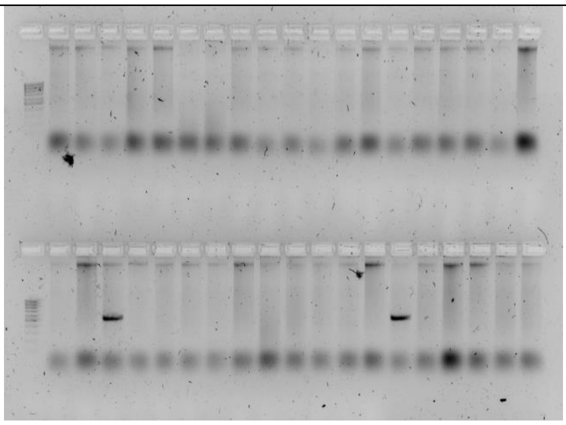

b

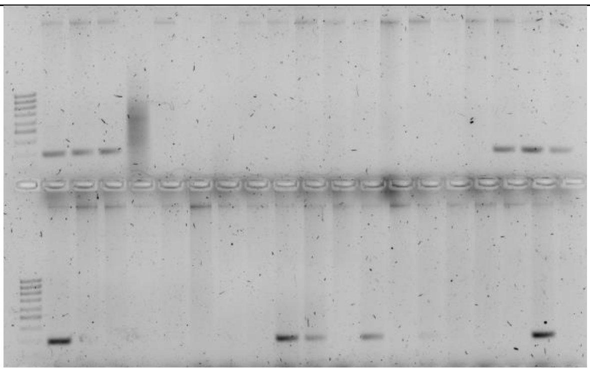

c

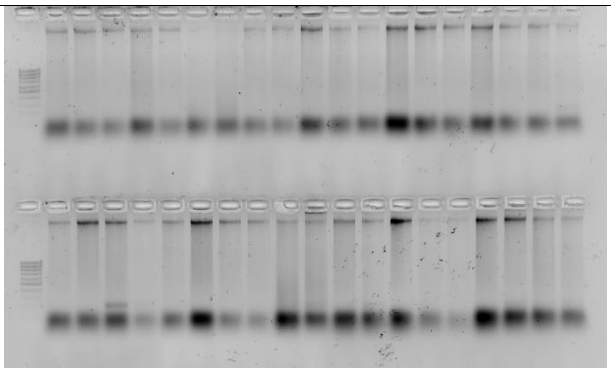

d

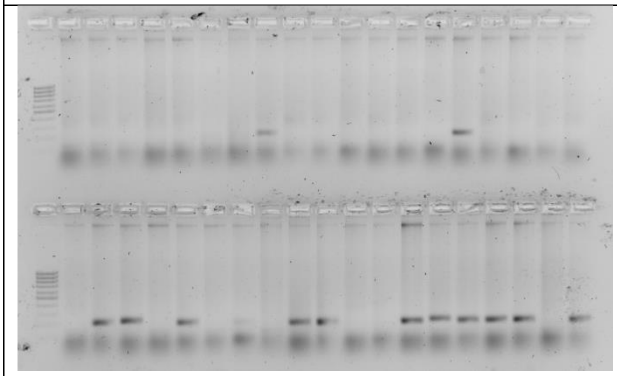

e

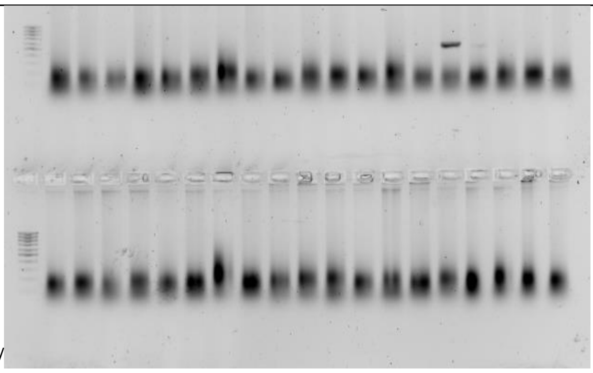

f

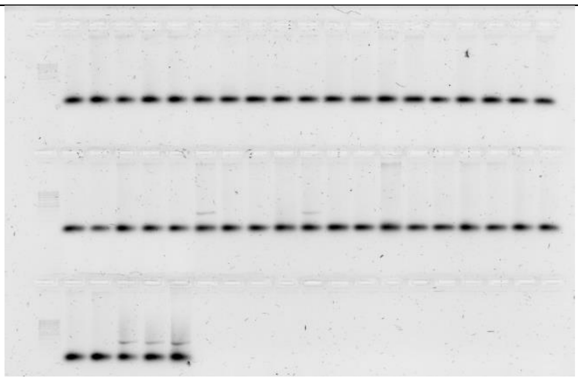

g

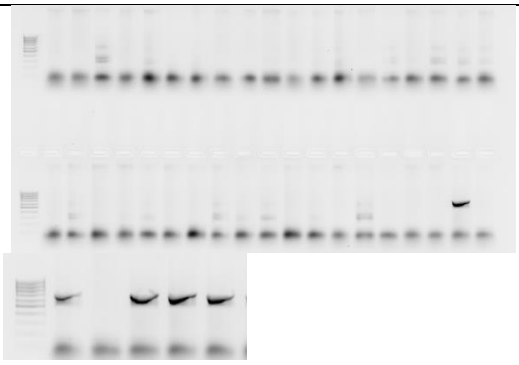

M 39 40 K K K

i

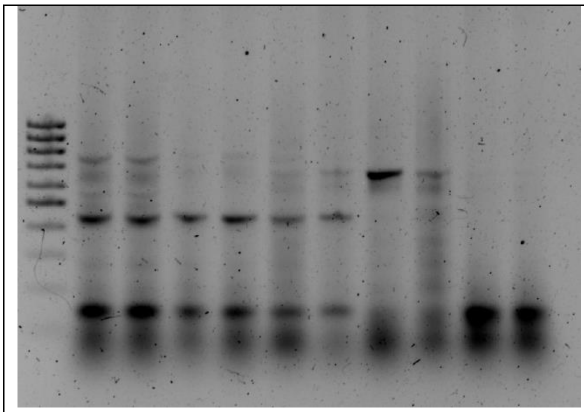

M 1 1 28 28 TcLr21 10 11 12 13

k

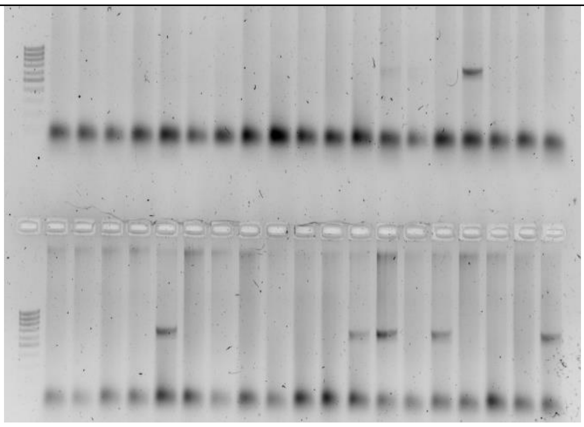

l

Figure. S1. Electrophoregram for markers: a) SCS265 (*Lr19*, *Sr25*), b) SCS5 (*Lr9*), c) F1.2245/Lr10-6/r2 (*Lr10*), d) Sr24#50 (*Lr24*, *Sr24*), e) SCM9 (*Lr26*, *Sr31*, *Yr8*, 1AL.1RS), f) STS638 (*Lr20*, *Sr15*), g) MF2/MR1r2 (Lr6Agi2), i) S13-R16 (*Lr66(Sp)*), k) Lr21F/R (*Lr21*), l) WR003 F/R (*Lr1*).

I, M - DNA Ladder 100 bp (Dialat), 1. Line 201m/22, 2. Line 334m/22, 3. Line 337m/22, 4. Line 55/08, 5. Line 143/09, 6. Line 42/93-09-1, 7. Line 1205-09-8, 8. Lutescens 54 190/09, 9. Lutescens 20 161/08, 10. Kudesnica, 11. Lutescence 2216, 12. Lutescence 2222, 13. Saru Akca 27, 14. Line 218/10, 15. PCIb12I 453, 16. PCIb12I I189, 17. Line 98-A-2, 18. Line 155-A-1, 19. Line 249-A-25.

II, M - DNA Ladder 100 bp (Dialat), 20. L-407/ChT, 21. L-6/SM, 22. L-235/PT, 23. KS 39/08-7, 24. KS 29/17y, 25. Lutescens 1485, 26. Lutescens 1510, 27. Lutescens 1535, 28. L373, 29. L447, 30. L2203, 31. L1353, 32. Kasibovskaya 2, 33. Lutescens 34-16, 34. Lutescens 205/12-5, 35. Lutescens 242/13-10, 36. Lutescens 74/16-1, 37. Pamyaty Tynina, 38. Zagadka

III, M - DNA Ladder 100 bp (Dialat),39. Erythrospermum 26464, 40. Line 1616ae14
